# Comparative Transcriptome Analysis Reveals Potential Molecular Regulation of Organic Acid Metabolism During Fruit Development in Late-Maturing Hybrid Citrus Varieties

**DOI:** 10.3390/ijms26020803

**Published:** 2025-01-18

**Authors:** Xiaoyu Tang, Mengqi Huang, Lijun Deng, Yixuan Li, Xiaojun Jin, Jiaqi Xu, Bo Xiong, Ling Liao, Mingfei Zhang, Jiaxian He, Guochao Sun, Siya He, Zhihui Wang

**Affiliations:** 1 College of Horticulture, Sichuan Agricultural University, Chengdu 611130, China; 2 Institute of Pomology and Olericulture, Sichuan Agricultural University, Chengdu 611130, China

**Keywords:** late-maturing hybrid citrus, fruit development, organic acid, transcriptome

## Abstract

Late-maturing hybrid citrus is a significant fruit that combines the best traits of both parents and is highly prized for its unique flavor. Not only can organic acids alter the flavor of citrus pulp, but they are also essential for cellular metabolism, energy conversion, and maintaining the acidbase balance in plant tissues. Although organic acids play a key role in the quality formation of citrus fruits, there is still insufficient research on the metabolic processes of organic acids in late-maturing hybrid citrus varieties. In this study, three late-maturing citrus varieties with different acidity levels, namely *‘Huangjinjia’* (HJ), *‘Kiyomi’* (QJ), and *‘Harumi’* (CJ), were selected to systematically investigate the metabolic regulation mechanism of organic acids in late-maturing citrus through transcriptome sequencing technology, combined with physiological and biochemical analyses. This study revealed gene expression differences related to organic acid synthesis and degradation. Through gene expression profiling, several genes closely associated with organic acid metabolism were identified, and a preliminary gene network related to the regulation of organic acid metabolism was constructed. The results showed that there were significant differences in the organic acid metabolic pathways between different varieties and growth stages of the fruit. Specifically, HJ had a higher TA content than QJ and CJ, primarily due to the significantly higher citric acid and malic acid contents in HJ compared to the other two varieties. Further analysis revealed that four gene modules showed a high correlation with the levels of major organic acids in the fruits. The genes involved in these modules are closely related to organic acid synthesis, degradation, and transport. Additionally, we also identified several key genes (*AS1*, *BZP44*, *COL4*, *TCP4*, *IDD10*, *YAB2*, and *GAIPB*) that might be involved in the regulation of organic acid metabolism. The functions of these genes could have a significant impact on the expression levels changes of enzymes related to organic acid metabolism. This study provides a foundation for exploring the intrinsic mechanisms regulating the organic acid content in late-maturing hybrid citrus fruits and contributes to the functional research of organic acids in late-maturing hybrid citrus and the molecular design of high-quality varieties.

## 1. Introduction

Citrus fruits are the most traded fruit internationally because they have a distinct flavor and are widely cultivated and sold due to their health-promoting qualities. Citrus hybrids between citrus fruits, oranges, or pomelos have surpassed traditional citrus types in popularity as fresh citrus variations because they have the best qualities, high nutritional contents, and ease of peeling inherited from both parent varieties [1]. The *‘Huangjinjia’* cultivar, a hybrid of *‘Chunxiang’* tangelo and *‘Kiyomi’*, features a distinctive copper coin mark at its base, possesses yellow flesh, emits a characteristic orange and pomelo aroma, and exhibits an average organic acid content of 1–1.1%. The *‘Kiyomi’* variety, a progenitor of *‘Huangjinjia’*, shows an average organic acid content of 0.8% [2]. In contrast, the *‘Harumi’* variety is noted for its robust flavor and has an organic acid content of 0.68%.

“High quality” has become essential for fresh fruits due to changes in people’s consumption conceptions and quality of life, and the flavor quality of citrus fruits is strongly affected by organic acids [3]. People’s sensitivity to sweetness is influenced by organic acids, and the sugar/acid ratio plays a critical role in deciding when to harvest fruit and what kind of fruit customers prefer [4]. Furthermore, components of the organic acid production pathway are crucial for several physiological functions, including plant growth, adversity tolerance, and intracellular pH homeostasis maintenance [4]. Depending on the variety and growth stage, the fruit’s organic acid types and ratios vary, and not all fruits have the same quantity of each acid [5]. When citrus fruits are fully ripe, 70–90% of the total organic acids are citric acid. Other organic acids are found in limited quantities in mature citrus fruits, with malic acid being the second most abundant [6,7]. The diversity and abundance of organic acids vary across different cultivars of fruits. Consequently, the key enzymes that influence organic acid accumulation will also differ [8]. The pyruvate pathway and sugar metabolism provide the essential building blocks for the synthesis of citric acid [9,10]. The synthesis of citric acid primarily occurs in the mitochondria of juice cells. After pyruvate enters the mitochondria to form acetyl-CoA, it enters the tricarboxylic acid cycle (TCA). This entry is facilitated by the action of enzymes such as citrate synthase (CS), aconitase (ACO), malate dehydrogenase (MDH), and isocitrate dehydrogenase (IDH) [11,12]. The created citric acid can be partially broken down to create energy and other metabolites, with the remainder being stored in liquid cells [4]. The cytoplasm of citrate is recognized to occur via three distinct pathways: the ATP-citrate lyase (ACL) pathway, the GS pathway, and the ACO-GABA pathway [12,13]. Glutamate decarboxylase (GAD), glutamine synthetase (GS), and ATP-citrate lyase (ACL) are the three main enzymes in the degradation pathway. Malic acid primarily transforms into oxaloacetate through the action of phosphoenolpyruvate (PEP), guided by two enzymes: PEP carboxylase (PEPC) and NAD-dependent malate dehydrogenase (NAD-cyt-MDH) within the cytoplasm [14]. Moreover, NADP-dependent malic enzymes (NADP-MEs) possess the ability to catalyze the reversible conversion of pyruvate into malic acid [15].

The build-up of organic acids has been associated with a variety of transcription factors in scientific studies. In the acidity analysis of Wenzhou citrus oranges [16], researchers discovered that the breakdown of citric acid is enhanced by the pathway *CitAco3-CitGS2-CitGDU1*, which is essential for the glutamine metabolic pathway. Additionally, *CitCHX* and *CitDIC*, two transport-related genes, are likely linked to citric acid degradation. In kiwifruit, the transcription factor *AcNAC1* activates *AcALMT1* expression by directly binding to its promoter. Moreover, *AcALMT1* functions as a transport protein that mitigates the build-up of citric acid [17]. The P3A-type ATPase *MdMa11* in apples regulates fruit acidity, and *MdESE* acts as a promoter for *MdMa11*, activating the gene to influence organic acid accumulation in fruit [18]. In strawberries, *FaMYB5* enhances citric acid levels by stimulating citrate synthase expression while inhibiting citrate degradation enzymes [19]. The transcription factor *MdbHLH3* directly manages *MdcyMDH* transcription during early apple fruit development, thereby controlling the synthesis and transport of malic acid [20].

The majority of current research on organic acids in citrus focuses on determining fruit composition at maturity, market value, and disease resistance, with fewer studies on the molecular mechanisms of organic acid degradation during the growth process of late-maturing *Citrus sinensis* fruits. In this study, three late-maturing citrus varieties with varying acidity were used as experimental materials to comprehensively analyze titratable acid (TA), organic acid composition and content, and key enzyme activities throughout the entire growth and development process. Based on the changes in organic acids, a key period of transcriptomic sequencing was performed to obtain transcriptome profiles. The study’s aims were as follows: (i) to investigate the physiological dynamics of organic acid metabolism, including component composition and content, as well as the dynamics of important enzyme activities; and (ii) identify major differentially expressed genes (DEGs) involved in organic acid regulation at various developmental stages in three late-maturing hybrid citrus cultivars. This study provides a theoretical basis for investigating the regulatory measures of different organic acid contents in fruits. It also offers a scientific foundation and valuable genetic resources for studying the fundamental characteristics of fruits, accelerating the development of low-acid varieties, and selecting and breeding new varieties.

## 2. Results

### 2.1. TA and Organic Acid Content in the Growth of Late-Maturing Hybrid Citrus Varieties with Different Acidities

**Figure 1:**
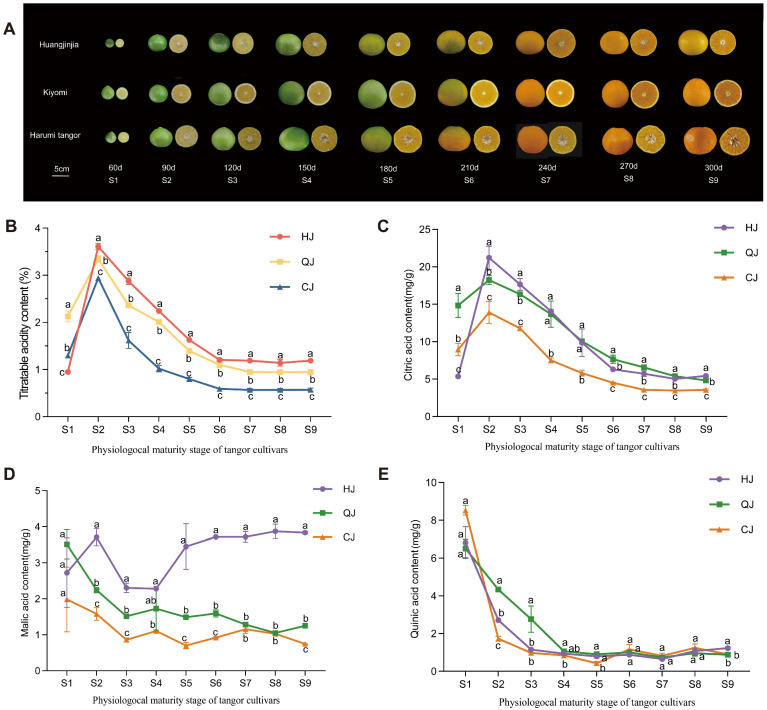
(**A**) Morphological changes during fruit development and ripening of the three cultivars (‘Hungjinjia’, ‘Kiyomi’ and ‘Harumi’). (**B**) TA content of citrus. Changes in citric acid (**C**), malic acid content (**D**) and quinic acid (**E**). Error bars indicate the standard error of total substance content, and different letters indicate signifi-cant differences in total content, *p* < 0.05.

The morphological traits of growth and development for the three late-maturing hybrid citrus varieties are illustrated in Figure 1A. The flesh of the fruit turns orange as it ripens. The titratable acidity content in these varieties fluctuates significantly throughout fruit growth. This process consists of two distinct phases (Figure 1B): a build-up of titratable acidity during the S1–S2 stages, followed by a gradual decline until stabilization after the S2–S9 phase. There were variations in the levels of titratable acidity present in the different varieties. Specifically, the titratable acidity content in HJ was 1.25 times greater than that in QJ and 2.11 times higher than that in CJ. These differences were significant (*p* < 0.05), indicating that there were significant differences in the metabolic processes of organic acid accumulation and degradation among the varieties.

In order to elucidate the trends in organic acid fractions in fruits of three citrus varieties after flowering, the contents of organic acid fractions in citrus fruit pulp at different stages were detected and analyzed using ultrahigh-performance liquid chromatography (HPLC). Based on the HPLC results, it was shown that a total of three organic acids with significant changes were detected: citric, malic, and quinic acids. The results showed that there were noticeable variations in the amounts of citric acid in the fruit as it grew, showing a pattern of first increasing and then decreasing, with the concentration peaking at stage S2 (Figure 1C). Notably, HJ exhibited significantly higher citric acid levels compared to QJ and CJ. At the near-maturity stage, the citric acid content of the HJ variety was 5.43 mg/g, significantly higher than the other two varieties, while the citric acid content of the CJ variety was the lowest, at 3.56 mg/g.

Regarding the variations in malic acid content (Figure 1D), the fruits of QJ and CJ all displayed a gradually declining trend in malic acid content, and the content of HJ displayed a rising, decreasing, and then increasing trend. In the S2 stage, the malic acid content of the HJ variety was the highest, reaching 3.71 mg/g. As the fruit matured, the malic acid content of QJ and CJ gradually decreased to 1.25 mg/g and 0.75 mg/g, respectively. However, the malic acid content of the HJ variety increased at the S4 stage and remained between 3.45 and 3.87 mg/g (Supplementary Table S1). All three varieties showed a slow and consistent decline in their quinic acid content (Figure 1E). During growth and maturity, the total acid fractions in HJ and QJ followed the same trend as titratable acid, increasing and subsequently decreasing, but the alterations in HJ were significant.

### 2.2. Determination and Analysis of Enzyme Activities Related to Organic Acid Metabolism in Fruit of Citrus Varieties

**Figure 2:**
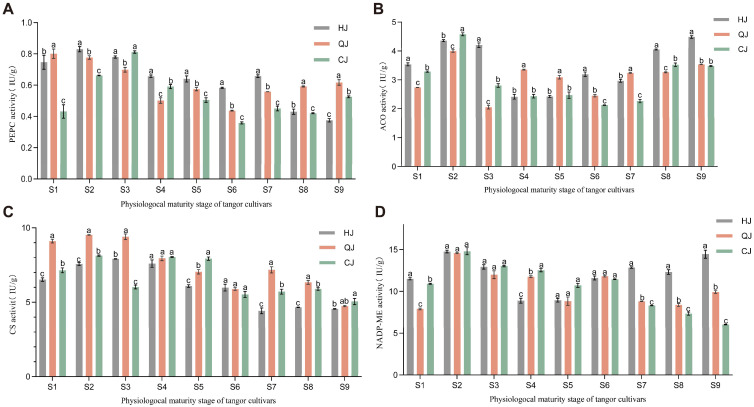
Changes in PEPC (**A**), ACO (**B**), CS (**C**), and NADP-ME (**D**) activities during citrus fruit development. Data are expressed as the mean of three biological replicates. Error bars indicate the standard error of total substance content, and different letters indicate signifi-cant differences in total content, *p* < 0.05.

The PEPC activity varied significantly throughout the fruit’s growth and maturity (Figure 2A). The trends of changes in PEPC enzyme activity in the three varieties were generally consistent. When the PEPC enzyme activity reached its peak, the enzyme activities of HJ, QJ, and CJ were 0.83 IU/g, 0.80 IU/g, and 0.81 IU/g, respectively. The PEPC enzyme activity steadily declined as the fruit grew older. HJ exhibited noticeably higher enzyme activity than the other two types in the majority of phases. The patterns of ACO activity in the three variations were not consistent (Figure 2B). With values of 4.36 IU/g, 4.00 IU/g, and 4.58 IU/g, respectively, HJ, QJ, and CJ reached the highest ACO activity in the S2 stage, which subsequently gradually decreased before increasing again during the S7–S9 stages. Figure 2C demonstrated that, during the growth of citrus fruits, the same trend of overall changes in CS enzymes could be observed. Enzyme activity variations between types were considerable throughout the early stages of fruit development (S1–S3), with QJ having significantly higher enzyme activities than HJ and CJ. However, after S4, these differences ceased to be significant. NADP-ME activity in citrus fruits of the three varieties did not differ significantly in terms of enzyme activity at the time of reaching the peak value (Figure 2D); however, the NADP-ME activity in HJ was significantly higher than that in QJ and CJ at late developmental stages (S7–S9).

### 2.3. Identification of DEGs Associated with Organic Acid Degradation Using Transcriptome Analysis

**Figure 3:**
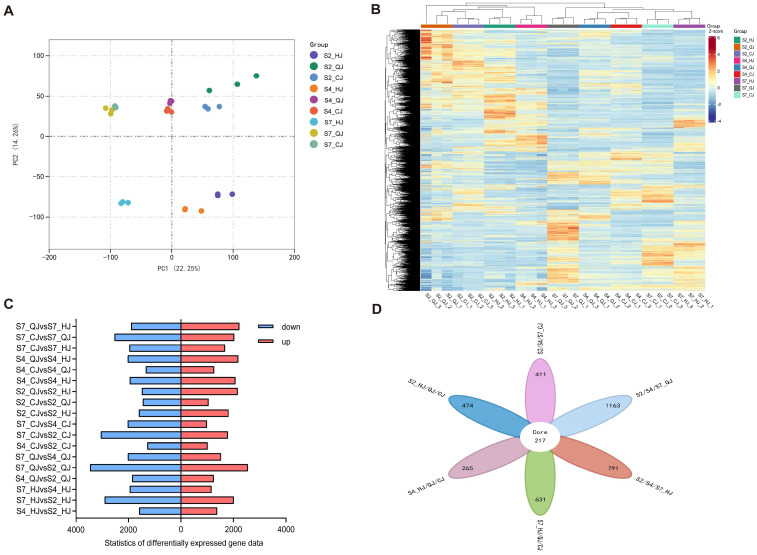
(**A**) Principal component analysis (PCA) of all samples. (**B**) Heatmap for hierarchical clustering based on different samples. (**C**) Number of DEGs in different DEG sets. (**D**) Venn diagrams of DEGs in all sample comparisons.

To explore potential key genes involved in the organic acid metabolism of late-maturing hybrid citrus fruits, high-throughput sequencing was performed on the constructed citrus fruit pulp transcriptome library using the Illumina platform. The transcriptome includes three varieties, with pulp samples taken at three developmental stages (S2, S4, and S7) for each variety. The TA content was highest at the S2 stage, while the TA content remained stable at the S7 stage. In this experiment, a total of 27 transcriptome datasets were acquired. Following sequencing, the raw datasets were filtered, and the distribution of GC content and sequencing error rates were examined. A total of 1,397,931,254 clean reads were extracted, with a clean base of 209.72 G. Each sample’s error rate was less than 0.03%, and the GC content varied from 44.2% to 45.52%, with Q30 > 93.79% and Q20 > 97.98% (Supplementary Table S2). The expression levels of 12,832 genes were quantified as FPKM values, and their distribution across the nine sample groups was visually represented through box-and-whisker plots (Supplementary Figure S1). To cluster the interactions between the nine groups of samples, principal component analysis (PCA) was performed using the FPKM values (Figure 3A). The outcomes revealed that PCA1 and PCA2 explained 22.25% and 14.26% of the total variance, respectively. The findings demonstrated that the transcript levels varied significantly between varieties, as evidenced by the three replicates of every sample clustering together and the samples of different varieties being separated from one another. Particularly, the QJ and CJ varieties at matching developmental stages, exhibited close interrelation, whereas the HJ variety was easily distinguished from the other two varieties at the same developmental level. The experiments illustrated that the developmental differences in HJ at this point were noticeably larger than those observed in the other two varieties. Furthermore, this study used hierarchical cluster analysis to divide the 31,740 genes into two branches in the heatmap (Figure 3B), which was found to be comparable to the principal component analysis results. All in all, these analyses demonstrated the reproducibility of the biological replicates of the samples.

The differential genes of the three late-maturing hybrid citrus varieties at S2, S4, and S7 were compared two at a time, and the statistical findings from this study are visually represented in the figure (Figure 3C). From the two-by-two comparison groups, the greatest number of DEGs was discovered in S7 vs. S2, with 6017 genes (2553 up-regulated genes and 3464 down-regulated genes) in S7_QJ vs. S2_QJ. Furthermore, when comparing the DEGs across different varieties within the same timeframe, it was evident that the number of DEGs was notably higher in HJ compared to the other varieties (QI and CJ). For instance, S4_CJ vs. S4_HJ exhibited 4025 DEGs (comprising 2077 up-regulated and 1948 down-regulated genes), whereas S4_QJ vs. S4_HJ showed 4212 DEGs (including 2184 up-regulated and 2028 down-regulated genes). In conclusion, S2 and S7 appear to be critical periods for the distinct alterations in the growth metabolism of late-maturing hybrid citrus varieties. During these time periods, notable metabolic variations were observed between HJ and the other two varieties.

In order to clarify the differential expression of the nine samples, their DEGs were identified by making Venn diagrams. First, a comparative analysis of various hybrid citrus varieties over the same time period (Supplementary Figure S2A) showed that, in any two-variety comparison, the S7 stage had the highest number of unique DEGs and the S2 stage had the highest number of shared DEGs among various varieties, totaling 2834. We also compared varieties at different periods (Supplementary Figure S2B) due to significant variations in organic acid composition and content at different stages of growth and development. It was revealed that QJ exhibited the most shared DEGs as fruit maturity progressed, with all varieties displaying the highest count of unique DEGs in a two-variety comparison at S2 vs. S7. Through the integration of information from the Venn diagrams generated in the previous analyses, a total of 3952 DEGs were identified across all transcriptome data. A total of 217 genes were differentially expressed among these DEGs between two groups of adjacent stages and the three fruit types at different stages of maturity (Figure 3D).

**Figure 4:**
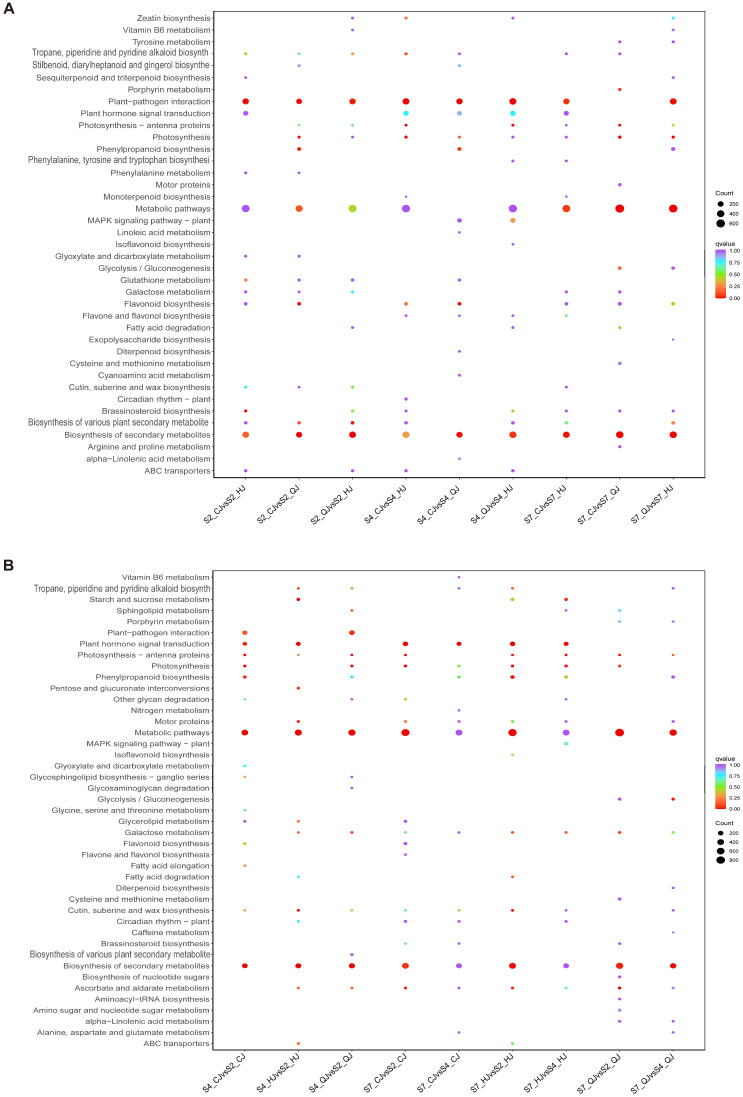
KEGG enrichment analysis of DEGs was depicted through bubble plots for different varieties during the same time period (**A**) and for the same variety across varying time periods (**B**).

To identify the biometabolic pathways linked to the DEGs obtained from the screening, enrichment analysis was performed against the reference pathways in the KEGG database. Bubble plots that show the KEGG enrichment analysis of DEGs across several kinds at a single timepoint (Figure 4A) and within the same variety at different timepoints (Figure 4B) are used to graphically illustrate the results. The KEGG analysis uncovered notable enrichment of metabolic pathways, biosynthesis of secondary metabolites, plant pathogen interactions, and four pathways related to plant hormone signal transduction.

### 2.4. Weighted Gene Co-Expression Network Analysis of RNA-Seq Data

**Figure 5:**
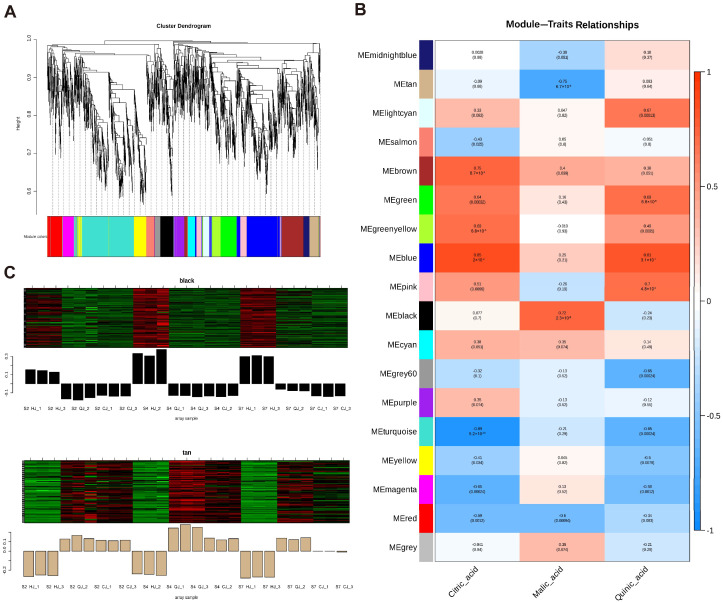
(**A**) The transcripts were partitioned into 18 modules by WGCNA based on gene correlation. The composite modules were then associated with organic acid components for correlation examination (**B**), focusing on the gene expression patterns of the MEtan and MEblack modules (**C**).

After sifting through the gathered gene FPKM values, a total of 27 transcripts were categorized into 18 modules through gene co-expression utilizing gene correlations (Figure 5A). We conducted correlation analysis of the three organic acid components with the expression patterns of each module to identify modules with phenotypic specificity to quinic, malic, and citric acids (Figure 5B). These included the following: the MEblack module had a positive correlation with malic acid (130 genes), the MEturquoise module had a negative correlation with citric acid and quinic acid (535 genes), the MEblue module had a positive correlation with citric acid and quinic acid (404 genes), and the MEtan module had a negative correlation with malic acid (93 genes). Upon completion of a correlation study of differentially expressed genes (DEGs) containing organic acid components, four modules were found to display significant correlations: MEblue, MEblack, MEturquoise, and MEtan. We chose four modules (MEblue, MEblack, MEturquoise, and MEtan) with substantial correlations to screen potential genes involved in organic acid metabolism based on the findings of the correlation analysis of these DEGs with organic acid components (Figure 5C; Supplementary Figure S2C).

**Figure 6:**
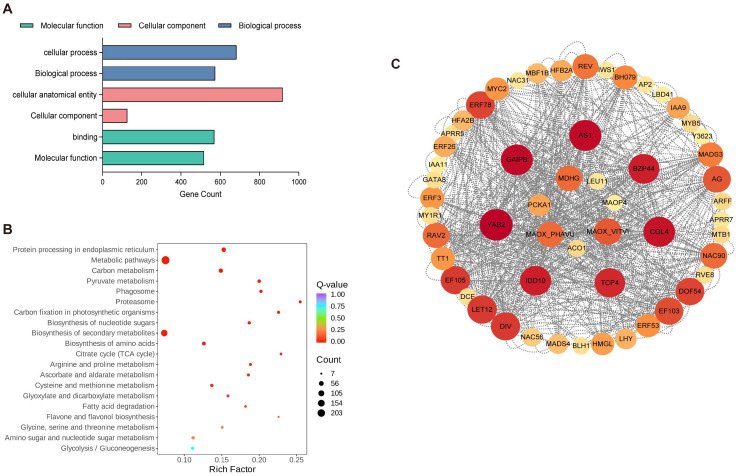
Subsequently, pivotal modules were identified for GO (**A**) and KEGG (**B**) analyses, followed by the construction of regulatory network maps (**C**).

GO enrichment analysis of the 1162 selected genes revealed significant enrichments in the cellular anatomical entity category, with additional enrichments in protein-containing complexes under the cellular component category. The 1162 chosen genes underwent GO enrichment analysis, which showed notable enrichments in the cellular anatomical entity category and further enrichments in protein-containing complexes under the cellular component category. The main annotations for molecular function included “binding” and “catalytic activity”, while the top annotations for biological process were “cellular process” and “metabolic process” (Figure 6A). “Metabolic pathways” and “biosynthesis of secondary metabolites” showed high enrichment, according to the KEGG analysis results. Additionally, it was shown that the pathways “glycolysis” and “citrate cycle (TCA cycle)” were similarly enriched (Figure 6B). These two pathways play a crucial role in cellular metabolism and are closely related to citric acid metabolism. Glycolysis and the TCA cycle are not only linked to each other but also work together to regulate intracellular citric acid concentration, which in turn affects the entire process of organic acid metabolism. Within the four modules analyzed, these genes are involved in various key enzymes that participate in important biological processes such as citric acid metabolism, carbohydrate metabolism, and amino acid metabolism. Specifically, the identified genes include the following: an *ACO* gene (CICLE_v10024840mg), which encodes *ACO*, a key enzyme in the citric acid cycle that catalyzes the conversion of aconitate to isocitrate; a *PEPC* gene (CICLE_v10025088mg), which encodes PEPC, involved in the synthesis of malic and citric acids; an *MDH* gene (CICLE_v10025945mg), which encodes MDH, an enzyme that catalyzes the reversible conversion between malate and oxaloacetate, participating in the regulation of the organic acid balance within cells; three *NADP-ME* genes (CICLE_v10007668mg, CICLE_v10007810mg, and CICLE_v10025125mg), which encode NADP-dependent malic enzymes (NADP-MEs), enzymes that decarboxylate malate to produce pyruvate, further participating in energy production and metabolic regulation; a *GAD* gene (CICLE_v10015017mg), which encodes GAD, an enzyme that plays a role in converting glutamate to GABA, an important adaptive mechanism in plants to cope with environmental stress; and a *CS* gene (CICLE_v10020218mg), which encodes CS, responsible for combining oxaloacetate with acetyl-CoA to form citric acid, the first key step in the TCA cycle. The enrichment of these genes provides important clues for further studying organic acid metabolism and its regulatory mechanisms, as well as valuable molecular information for the in-depth analysis of plant metabolic networks. In this study, 55 transcription factors co-expressed with structural genes in the module were screened, and a regulatory network was built to further illustrate the transcription factors that might control organic acid metabolism. As shown in Figure 6C, these include 6 *AP2/ERF-ERF*, 4 *MYB*-related, 4 *NAC*, 3 *bHLH*, and 29 other family genes. Among them, *IAA9*, *TT1*, *AS1*, *REV*, *LET12*, *EF105*, *TCP4*, *IDD10*, *AG*, *GAIPB*, *EF103*, and *BZP44* were strongly correlated with structural genes (>0.9). Intramolecular hub genes, identified as *AS1*, *BZP44*, *COL4*, *TCP4*, *IDD10*, *YAB2*, and *GAIPB*, were likely associated with the control of organic acid metabolism.

### 2.5. Organic Acid Metabolic Pathways Involved in the Development of Late-Maturing Hybrid Citrus

**Figure 7:**
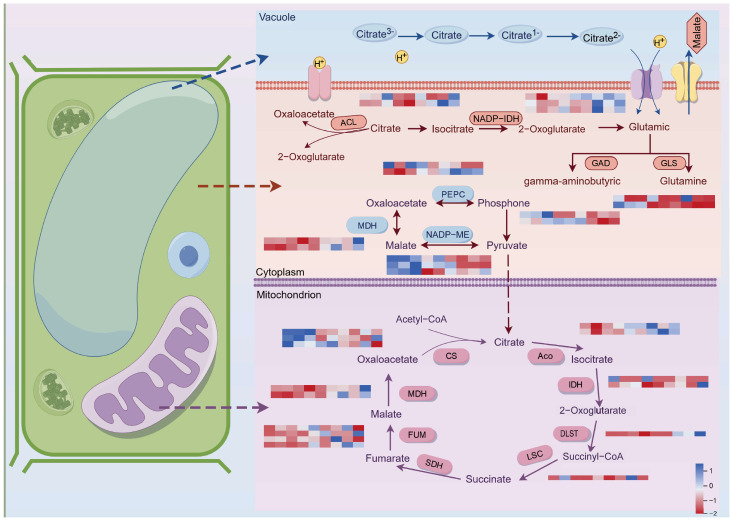
A heatmap that displays the genes linked to the metabolic processes in charge of the production and degradation of malic and citric acids. Gene expression is displayed in heatmaps based on mean FPKM. Azure indicates high expression, and pink indicates low expression.

Citric acid and malic acid were the primary organic acid constituents in citrus, based on prior results from organic acid contents. PEPC catalyzes the synthesis of oxaloacetate from phosphoenolpyruvate in the cytoplasm, which subsequently passes through the TCA cycle in the mitochondria to produce citric acid [21,22]. Within the cytoplasm, oxaloacetate generated by PEPC from phosphoenolpyruvate can also be converted to malic acid through the activity of MDH, or it can undergo further processing in the mitochondria via the TCA cycle to yield malic acid [23]. Additionally, malic acid can be formed from malic acid through the action of ME [24]. It was discovered that 26 and 5 structural genes, respectively, are involved in the metabolism of citric acid and malic acid throughout their synthesis and metabolism. Based on their FPKM values (log_2_(FPKM)), the gene expression regulatory networks in the flesh of the three late-ripening hybrid citrus fruits were mapped (Figure 7). *MDH* genes (CICLE_v10016114mg and CICLE_v10025945mg) showed an overall progressive increase in expression as the fruit ripened; among them, *MDHM* (CICLE_v10016114mg) exhibited a decrease and subsequent increase in FPKM in HJ, which was compatible with the trajectory of malic acid content. The expression of two *CS* genes (CICLE_v10005099mg and CICLE_v10020218mg) was downregulated, with CICLE_v10020218mg showing lower expression than HJ and QJ in all three CJ stages. HJ expression was consistently higher. The expression of a *PEPC* gene (CICLE_v10025088mg) was higher in HJ varieties and relatively lower in CJ and QJ varieties. The expression of one *GAD* gene (CICLE_v10015017mg) on the citrate degradation pathway was gradually weakened, and the expression of HJ was significantly lower than that of QJ and CJ at the same timepoint, while the expression of one *GLS* gene (CICLE_v10003183mg) was gradually up-regulated, with *GLS* gene (CICLE_v10003183mg) expression being significantly higher than that of HJ and QJ in CJ during the same period.

### 2.6. Validation of RNA-Seq Data Using qRT-PCR

**Figure 8:**
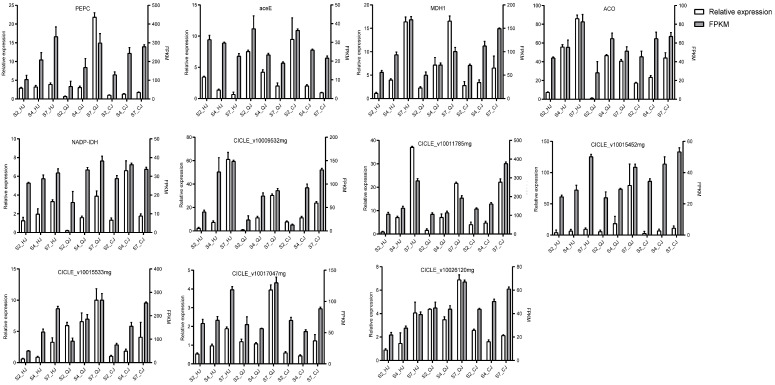
Validation of the differential expression of 11 genes using qPCR.

To evaluate the correctness of the RNA-Seq data, the expression levels of 11 randomly selected genes from the pathway associated with organic acid metabolism were assessed across all samples. The findings revealed concordance between the FPKM values derived from transcriptome sequencing results (Figure 8) and the qRT-PCR results. These results indicated the reliability of the transcriptome sequencing data for further investigations. Additionally, during the ripening process, the expression levels of *MDH* (CICLE_v10025945mg) and *PEPC* (CICLE_v10025088mg) exhibited a consistent increase, whereas *aceE* (CICLE_v10020224mg) showed a decrease in expression.

## 3. Discussion

Citrus fruits’ organic acids are crucial markers of fruit quality and serve as precursors for a variety of metabolic processes, including glycolysis, the TCA cycle, and gluconeogenesis [25]. Aligned with the empirical results presented by Yang et al. [26], the concentration of organic acids in the conducted study exhibited a pattern of increase, subsequent decrease, and eventual stabilization over the course of the experiment. At phase S9, HJ exhibited the most elevated levels of titratable acidity, succeeded by QJ and CJ in descending order of concentration. When citrus fruits are ripe, citric acid usually predominates as an organic acid, followed by malic acid [27]. The levels of citric, malic, and quinic acids exhibited notable variations across the growth stages of the three citrus varieties under examination. Among them, citric acid all showed an increasing and then decreasing trend, and HJ was significantly higher than QJ and CJ, which may be one of the reasons for the higher content of titratable acid in HJ than other varieties. The malic acid content fluctuated differently among the varieties, with QJ and CJ displaying a gradual decrease overall, which is consistent with the findings from Sun’s research [8,28]. However, the content of HJ showed a trend of increasing, then decreasing, and then increasing again, and as the fruit matured, the content of QJ and CJ gradually decreased to 1.25 μg/g and 0.75 μg/g, but the malic acid of HJ was maintained at 3.45–3.87 μg/g, which was 2.76 times and 4.60 times higher than that of QJ and CJ, and it was the main reason for the acid content of HJ being higher. Quinic acid levels displayed a consistent decline with minimal variance between the varieties as they ripened [1,29].

During fruit development, changes in organic acid content are accompanied by changes in the activities of the corresponding enzymes, such as CS, ACO, PEPC, and NADP-MEs, all of which influence the accumulation of organic acids. PEPC enzyme and NADP-MEs enzyme play a role in malate synthesis in the cytoplasm, whereas CS enzyme and ACO enzyme together influence citric acid accumulation. Previous experiments have shown that CS regulates fruit citric acid accumulation to some extent [30]. The results of the present study showed that CS enzyme was significantly and positively correlated with citric acid, and the trends in both were consistent, but CS activity showed some differences in different flavor citrus, which was in agreement with the results of Gao et al. [31]. Aco enzyme is closely related to citric acid degradation [30], and in this experiment, CS activity and ACO enzyme were found to be closely related to citric acid degradation. The trends in citric acid content and ACO enzyme activity were consistent during pre-fruit development and showed opposite trends during ripening. Just as Chen et al. [32] found that treatment enhanced the expression of Cit Aco3 but not Cit Aco1 and Cit Aco2 in citrus fruits, this indicates that there is still a lack of clear understanding of the mechanism of Aco genes in the degradation of citric acid in citrus fruits. At the late stage of citrus fruit development, the changes in malic acid content and NADP-ME activity in HJ, QJ, and CJ were consistent with the trend in changes in NADP-ME activity, and the rise in NADP-ME activity at the late stage led to the degradation of malic acid, but the total amount of malic acid in the fruit was likely increased due to the rate of synthesis being greater than the rate of degradation. This result is similar to that of a previous study on pineapple [31]. Therefore, we hypothesize that differences in NADP-ME activity during the later stages of citrus fruit development are representative of the main reasons for the differences in malic acid content in different varieties.

Numerous advancements have been achieved in elucidating the molecular mechanisms underlying citrus fruit, with significant genes associated with fruit quality being documented. Significant discoveries highlight the role of *CsPH8* in regulating citric acid storage within vesicles by encoding a plasma membrane H^+^-ATPase [33]. Citrus WRKY transcription factors (*CsWRKY3*, *CsWRKY47*, and *CsWRKY46*) have demonstrated clear involvement in the metabolism of citrus sugar and acid [34]. The *CitHsfA7* transcription factor induces HAT by binding to *CitAco3*, thereby promoting citrus fruit citric acid degradation in citrus fruits [35]. In the context of pear fruits, *PpWRKY44* and *PpABF3* have demonstrated the ability to modulate malic acid levels, with *PpWRKY44* activating malic acid accumulation by directly binding to the promoter of the malic-acid-related gene *PpALMT9* [36]. However, the molecular mechanisms behind the metabolism of organic acids in late-maturing hybrid citruses are still being investigated. Thus, in order to determine the organic acid metabolic pathway in late-maturing hybrid citrus, we examined 27 RNA-Seq samples. Our analysis of the data revealed that the metabolic process was controlled by a complicated network.

The overall concentration of organic acids found in fruit is contingent upon four primary regulatory mechanisms governing acid production, catabolism, utilization, and distribution [37]. The TCA cycle plays a crucial role in cellular energy metabolism and the oxidation of pyruvate within the mitochondria to facilitate ATP synthesis, leading to the direct production of predominant citrus organic acids like citric acid and malic acid [38]. Research has indicated the significant involvement of key enzymes, including PEPC, ME, MDH, CS, IDH, and ACO, in the glyoxylate cycle. Transcriptome analysis of late ripening hybrid citrus fruit flesh identified 20 differentially expressed genes responsible for controlling various enzymes associated with the organic acid biosynthetic pathway in the TCA cycle, such as *PEPC* (CICLE_v10000510mg and CICLE_v10025088mg), *MDH* (CICLE_v10016114mg and CICLE_v10025945mg), and *CS* (CICLE_v10005099mg, CICLE_v10020042mg, and CICLE_v10020218mg), among others. Nine genes that exhibited differential expression were pinpointed, and these are associated with the enzymes implicated in the citrate degradation pathway. Among these genes were *ACL* (CICLE_v10031633mg and CICLE_v10004572mg), alongside several others. The genes encoding enzymes responsible for metabolic processes in fruit development exhibit varying expression patterns throughout different stages, influencing the levels of enzyme activities involved in the build-up or decomposition of organic acid [39,40]. The expression of *CS* (CICLE_v10020218mg) in CJ was lower than that observed in HJ and QJ, which could diminish the activity of the CS enzyme in CJ, resulting in decreased citric acid production. We found that the expression level of *PEPC* (CICLE_v10025088mg) was significantly higher in HJ varieties than in CJ and QJ varieties. The high expression of this gene may have promoted the activity of PEPC enzyme, which accelerated the malic acid synthesis process. PEPC enzyme a key enzyme in the malic acid synthesis pathway; therefore, it is hypothesized that the difference in the expression of *PEPC* (CICLE_v10025088mg) may be one of the molecular mechanisms for the higher accumulation of malic acid in HJ varieties. The expression of *GLS* (CICLE_v10003183mg) was higher in CJ than in HJ and QJ, and the expression in HJ was significantly lower than that of QJ and CJ at the same timepoint, and this difference in expression suggests that the GLS gene may have different regulatory effects on citric acid metabolism among different varieties. In particular, the elevated GLS enzyme activity may promote the citric acid degradation pathway during the citric acid degradation process, leading to a decrease in citric acid content. High expression of this gene in CJ varieties may lead to enhanced citric acid degradation and thus reduce citric acid accumulation, while low expression of this gene in HJ varieties may slow down the process of citric acid degradation and thus favor citric acid accumulation. Therefore, the higher citric acid content in HJ varieties may be closely related to the low expression of the GLS gene. In addition, the differences in the expression levels of the GLS gene across varieties may explain, to some extent, the variation in citric acid accumulation, further suggesting that the GLS gene may play an important regulatory role in the relationship between citric acid metabolism and variety characteristics. The pattern of malic acid content in HJ was in line with the FPKM of *MDHM* (CICLE_v10016114mg) in HJ, which first decreased and then increased. This gene was thought to facilitate the synthesis of malic acid, and the noteworthy differences in its expression in the later stages of HJ, QJ, and CJ development may be one of the causes of the higher malic acid content in HJ fruits opposed to the other two varieties. On top of that, while it is expected that metabolites from the TCA cycle would diminish as the fruit ripens, certain genes related to this cycle demonstrate increased expression levels, possibly as a result of heightened post-transcriptional and post-translational control mechanisms (Figure 7). A decrease in the amount of metabolites in the cell at the end of the cycle may result from the corresponding metabolites being quickly transported to the downstream metabolic pathways and the rate of depletion being faster than the rate of synthesis [41]. This could be explained by the cycle’s enhanced post-transcriptional and post-translational regulation [1].

**Figure 9:**
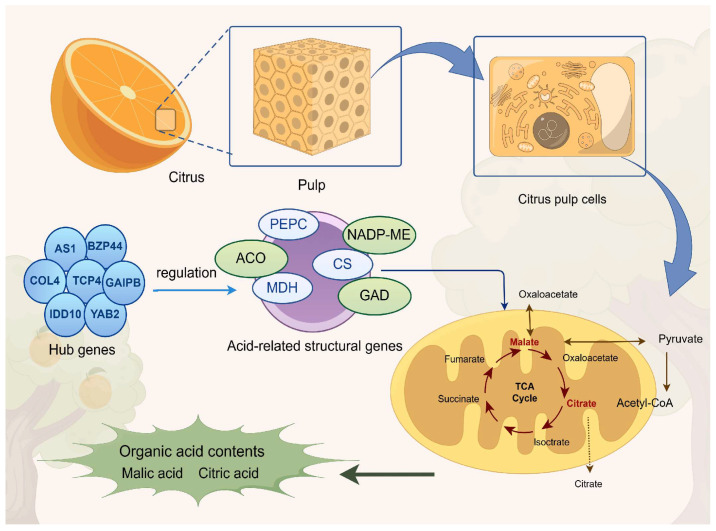
An organic acid metabolism model for late-ripening hybrid citrus is proposed. During fruit development, transcription factors and structural genes control the synthesis of organic acid components, as demonstrated by the identification of hub genes in trend analysis, WGCNA, heat maps, and transcriptome analysis.

Based on the regularity of their expression patterns, WGCNA can cluster genes that exhibit similar patterns of expression and organize the very comparable genes into modules. Several structural genes, transcription factors, organic acid transporter proteins, and other putative regulators linked to acid metabolism were investigated using WGCNA. Numerous inquiries have underscored the pivotal significance of transcription factors in orchestrating the build-up of organic acids within citrus fruits. CitERF13, for instance, rises in proportion to CitVHA abundance and encourages citrus fruits and Arabidopsis thaliana to have higher citric acid contents [42]. Similarly, the *MdMa7* (MDH1) promoter controls the binding capacity of the transcription factor MdbHLH74, which is a crucial gene for malic acid synthesis and, consequently, affects the amount of malic acid in apple fruits [11]. In the conducted study, all transcription factors within the 27 transcriptomic datasets were meticulously classified into 18 distinct modules using WGCNA. The study revealed a noteworthy association between the MEblue module and citric and quinic acids, indicating a positive correlation. Similarly, the MEblack module demonstrated a substantial positive relationship with malic acid. Conversely, the MEturquoise and MEtan modules showed a significant negative correlation with these three organic components. Notably, a number of transcription factors that were co-expressed with structural genes were identified within these highly correlated modules. *AS1*, *BZP44*, *COL4*, *TCP4*, *IDD10*, *YAB2*, and *GAIPB* were highlighted as potential candidates associated with the regulation of organic acid metabolism. These genes may play a significant regulatory role in organic acid metabolism. They may have an influence on the production and transformation of organic acids by controlling the activity of important enzymes through co-expression with these genes. Additionally, the citric acid cycle, the metabolism of carbohydrates and amino acids, and other metabolic pathways that are crucial for energy production, environmental adaptation, and plant growth and development may also be regulated by these genes. The reliability of the transcriptome data was confirmed by analyzing the expression of 11 genes in 3 types at 90 d (S2), 150 d (S4), and 240 d (S7) using qRT-PCR. We established a model to describe how hub genes and pertinent transcription factors affect organic acids in late-maturing citrus based on the findings of this investigation (Figure 9). The roles of these genes in the metabolism of organic acids still require further research.

## 4. Materials and Methods

### 4.1. Plant Materials and Sampling

For this study, we selected three late-maturing citrus varieties: *‘Huangjinjia’* (HJ), *‘Kiyomi’* (QJ), and *‘Harumi’* (CJ). Sampling began 60 days post-bloom and was carried out every 30 days at intervals of 60 days (S1), 90 days (S2), 120 days (S3), 150 days (S4), 180 days (S5), 210 days (S6), 240 days (S7), 270 days (S8), and 300 days (S9) following bloom. We established three replicates in plots containing three trees for each variety. From each tree, we randomly selected ten healthy and appropriate fruits from four different directions. Fruits from the three types were quickly peeled to separate the skin and pulp, with the pulp used as the experimental sample. A portion of the fresh pulp was juiced to measure titratable acidity (TA %) and organic acid content. The remaining pulp was quickly frozen and processed to preserve its molecular integrity for future transcriptome sequencing.

### 4.2. Assessment of Titratable Acidity, Organic Acid Content, and Enzyme Activity

The titratable acidity (TA) of the juice was assessed by taking 5 mL of the sample, adding 1–2 drops of phenolphthalein indicator, and titrating with sodium hydroxide until a stable pink color appeared, persisting for 5 s. The volume of NaOH used was noted, and the experiment was replicated thrice in a standalone manner for every individual sample [25].

For determination of organic acid content, the sample weighing 1.0 g was meticulously ground and mixed with 4 mL of pre-cooled 0.2% phosphoric acid (pH = 2.6). Subsequently, ultrasonic extraction was conducted for a duration of 20 min. The extracted sample was then stored at 4 °C, centrifuged at 10,000 rpm for 20 min, and the supernatant was transferred to a centrifuge tube. Then, 4 mL of 0.2% phosphoric acid was added into the precipitate, and the supernatant was centrifuged under the previous conditions and filtered into a sample bottle. After centrifuge under the above conditions, the supernatant was mixed with the previous one, fixed to a final volume of 10 mL, and filtered into the injection vial. Chromatographic separation was conducted using an Agilent 1260 High-Performance Liquid Chromatography (HPLC) system manufactured by Agilent Technologies (Santa Clara, CA, USA). A C18 column measuring 250 mm in length and 4.6 mm in diameter, packed with 5 μm particles, was employed for the separation process, which was carried out at a temperature of 25 °C. The experimental setup entailed the utilization of a 20 μL injection volume, employing a mobile phase composed of 3% aqueous methanol and 97% 0.2% phosphoric acid in water, flowing at a rate of 0.8 mL/min, while monitoring detection at a wavelength of 210 nm [43].

Enzyme activities were determined using ELISA kits for plant PEPC, CS, ACO, and NADP-MEs, obtained from Chongqing Bonoheng Biotechnology Company (Chongqing, China), following the manufacturer’s instructions.

### 4.3. RNA Library Preparation and Analysis of the Differentially Expressed Genes (DEGs)

The transcriptome consisted of pulp samples from three varieties and three timepoints (90 d (S2), 150 d (S4), and 240 d (S7)), with three biological replicates at each timepoint, for a total of 27 samples. High-fidelity RNAs harboring polyadenylated tails were selectively captured using Oligo(dT) magnetic beads, with subsequent utilization of these enriched RNAs in the construction of libraries. The RNA was fragmented into short segments facilitated by a buffer, serving as the template for subsequent processes. The first- and second-strand cDNAs were synthesized, and the double-stranded cDNA was purified using DNA purification beads. The cDNA fragments with a length of 370~420 bp were selected from the purified double-stranded cDNA, and then PCR enrichment was performed to obtain the final cDNA library. The PCR products underwent purification utilizing the AMPure XP system. *Citrus clementina* (citrus orange) genome as the reference genome. The alignment of high-quality sequencing data to a known reference genome was executed through the utilization of HISAT2 [44] in order to derive positional data with respect to the reference genome or gene, along with details pertaining to sequence attributes unique to the particular sample under investigation. Gene relative expression levels were measured as the expected number of fragments per kilobase fragment (FPKM) of transcript sequence fragments sequenced per million base pairs. Differential expression analysis was carried out using DESeq2 [45,46] on samples featuring biological duplicates to ascertain a collection of genes that displayed differential expression between distinct biological conditions, meeting the criteria of |log2Fold Change| > = 1 and FDR < 0.05. Kyoto Encyclopedia of Genes and Genomes (KEGG) [47] and Gene Ontology (GO) [48] were used to organically combined gene information with high-level functional information and protein function information to provide systematic analysis of big data generated by genome sequencing and other high-throughput experimental techniques.

### 4.4. Co-Expression Network Analysis

Weighted gene co-expression network analysis (WGCNA) clusters genes in the same module on the same color clustering tree with genes that have similar expression variations in a physiological process or in various tissues. The expression data were filtered using R language, with a filtering threshold of 0.85 to divide the different modules and perform hierarchical clustering analysis, and the clustering results are visualized as branching in the tree, where different gene modules are represented in different colors. Subsequently, the gene modules related to different kinds of organic acids were constructed, the genes of interest were identified from the key modules and the functions of some of the genes were predicted.

### 4.5. Quantitative Real-Time PCR (qRT-PCR) Analysis

Using Primer Premier5 software, we designed primers targeting specific genes involved in organic acid metabolism or related pathways (Supplementary Table S3). These primers were synthesized by Tsingke Biotech, Beijing, China. Using reverse transcriptase, we reverse-transcribed the isolated RNA (Mei5bio, Beijing, China). Subsequently, we performed qRT-PCR using SYBR qPCR Mix (Tsingke, Beijing, China). A Bio-Rad fluorescence quantitative PCR instrument facilitated the detection of the reactions. Relative gene expression values were calculated using the 2^−△△Ct^ method, with the citrus Actin gene as the internal reference gene [49].

### 4.6. Statistical Analysis

SPSS Statistics (19, IBM) was used for significance analysis, and Excel (2016, Microsoft), GraphPad Prism (10.1.2, GraphPad Software) and Adobe Illustrator (2020, Adobe) were used for graphing.

## 5. Conclusions

In this study, three late-maturing hybrid citrus varieties with different acidity were selected, the dynamic characteristics of organic acid metabolism at different growth stages were revealed by analyzing the changes in the content of organic acids in the different late-maturing hybrid citrus varieties, and significant differences in the organic acid fractions were found, among which the content of titratable acid in HJ was significantly higher than that of QJ and CJ because of the higher citric and malic acid contents in HJ, and most of all, because of the significantly higher content of malic acid compared to the other two varieties. Further using transcriptome sequencing technology combined with physiology and biochemistry, four gene modules were found to show a high correlation with the content of major organic acids in fruits, and some key genes closely related to the organic acid synthesis and degradation process were screened in these modules (*AS1*, *BZP44*, *COL4*, *TCP4*, *IDD10*, *YAB2*, and *GAIPB*), which may be important for the function of the genes. The functions of these genes may have a significant impact on the expression changes in enzymes related to organic acid metabolism, and a preliminary gene network related to the regulation of organic acid metabolism has been constructed. This study not only provides new perspectives on the regulation of organic acid metabolism, but also lays the foundation for improving the flavor, texture, and nutritional value of fruits through molecular breeding techniques in the future.

## Data Availability

The datasets presented in this study can be found in online repositories. The RNA-seq datasets can be found at: https://dataview.ncbi.nlm.nih.gov/object/PRJNA1147929, accessed on 16 January 2025.

## Supplementary Materials

The following supporting information can be downloaded at: https://www.mdpi.com/article/10.3390/ijms26020803/s1.
