# Supplementary material for: Comparative Transcriptome Analysis Reveals Potential Molecular Regulation of Organic Acid Metabolism During Fruit Development in Late-Maturing Hybrid Citrus Varieties
Source: Int J Mol Sci. 2025 Jan 18;26(2):803. doi: 10.3390/ijms26020803 (PMC11765802; doi:10.3390/ijms26020803)
Supplement: Supplementary file 1 [file ijms-26-00803-s001.zip › 2Supplementary figures.pdf]

International Journal of
Molecular Sciences

MDPI

Supplementary figures

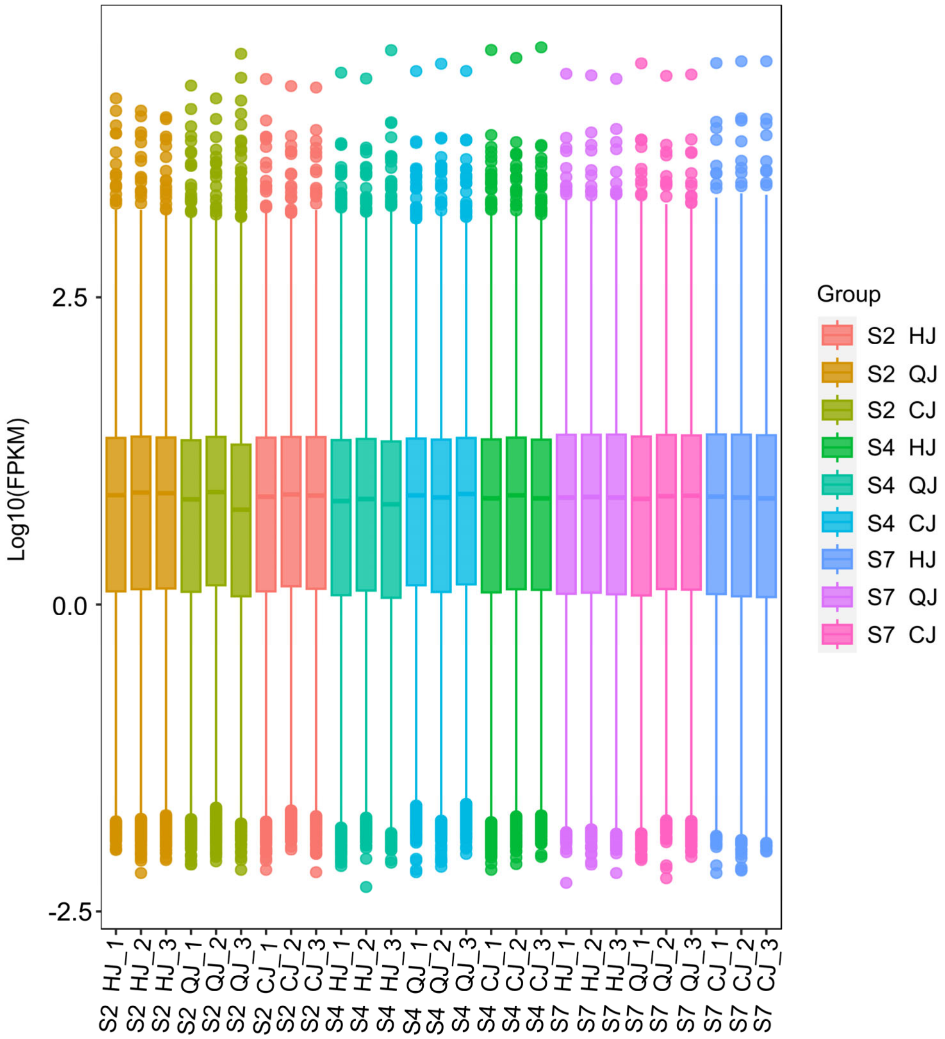

Supplementary Figure S1 : Box-and-line plot showing the distribution of gene expression levels in nine groups of samples.

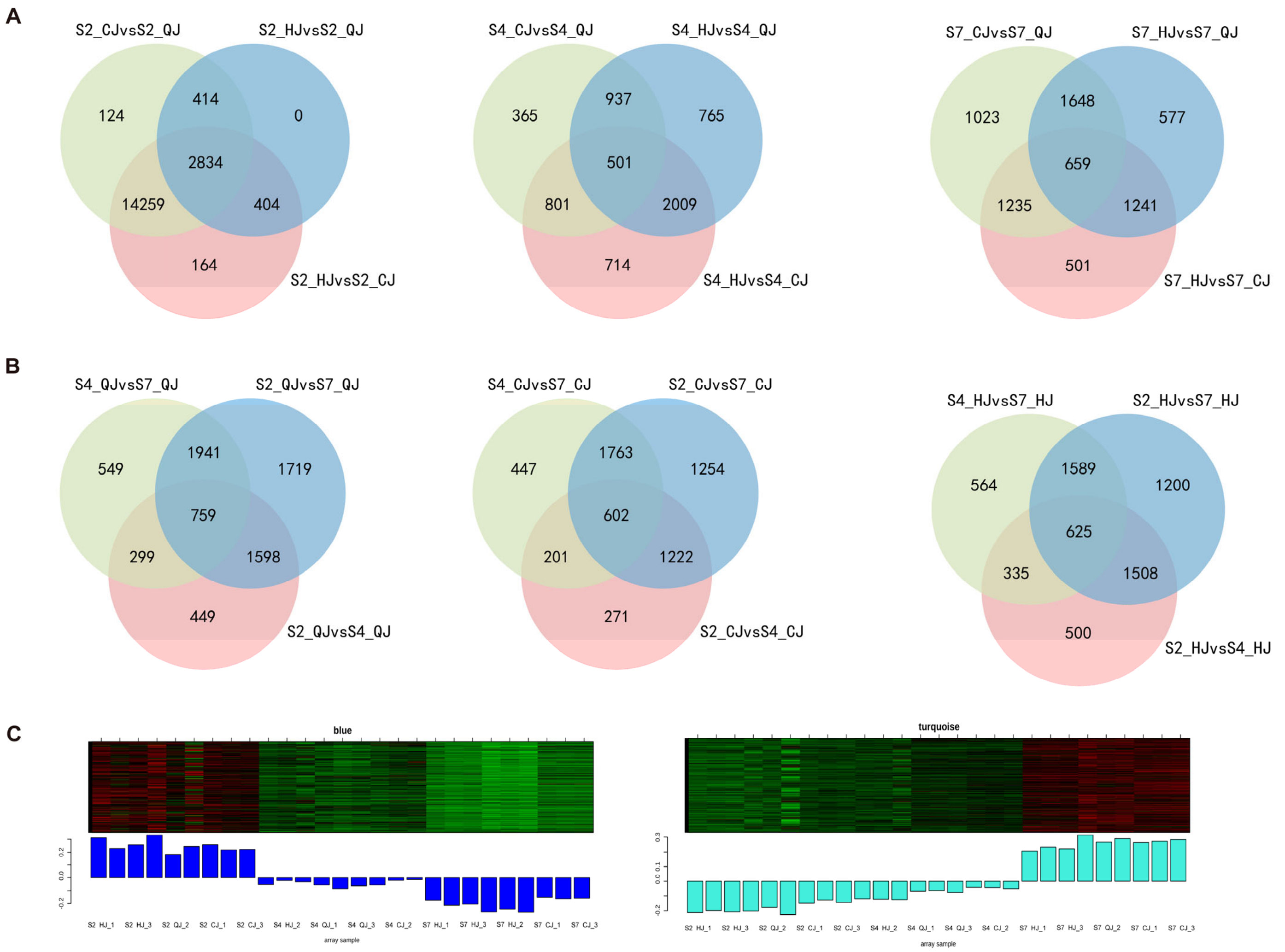

Supplementary Figure S2 : (A) Venn diagrams of three varieties in the S2, S4 and S7 period. (B) Venn diagrams of three critical periods in HJ, QJ, CJ varieties. (C) Two key modules of MEblue, MEturquoise.
